# Gold as a Possible Alternative to Platinum-Based Chemotherapy for Colon Cancer Treatment

**DOI:** 10.3390/cancers11060780

**Published:** 2019-06-05

**Authors:** Inés Mármol, Javier Quero, María Jesús Rodríguez-Yoldi, Elena Cerrada

**Affiliations:** 1 Department of Pharmacology and Physiology, University of Zaragoza, CIBERobn, IIS Aragón IA2, 50013 Zaragoza, Spain; ines.marmol9@gmail.com (I.M.); javierquero94@gmail.com (J.Q.); 2 Deparment of Inorganic Chemistry, Instituto de Síntesis Química y Catálisis Homogénea-ISQCH, University of Zaragoza-CSIC, 50009 Zaragoza, Spain

**Keywords:** colorectal cancer, gold(I) complexes, thioredoxin reductase, reactive oxygen species, gold nanoparticles, theragnosis, photodynamic therapy, photothermal therapy, radiotherapy

## Abstract

Due to the increasing incidence and high mortality associated with colorectal cancer (CRC), novel therapeutic strategies are urgently needed. Classic chemotherapy against CRC is based on oxaliplatin and other cisplatin analogues; however, platinum-based therapy lacks selectivity to cancer cells and leads to deleterious side effects. In addition, tumor resistance to oxaliplatin is related to chemotherapy failure. Gold(I) derivatives are a promising alternative to platinum complexes, since instead of interacting with DNA, they target proteins overexpressed on tumor cells, thus leading to less side effects than, but a comparable antitumor effect to, platinum derivatives. Moreover, given the huge potential of gold nanoparticles, the role of gold in CRC chemotherapy is not limited to gold(I) complexes. Gold nanoparticles have been found to be able to overcome multidrug resistance along with reduced side effects due to a more efficient uptake of classic drugs. Moreover, the use of gold nanoparticles has enhanced the effect of traditional therapies such as radiotherapy, photothermal therapy, or photodynamic therapy, and has displayed a potential role in diagnosis as a consequence of their optic properties. Herein, we have reviewed the most recent advances in the use of gold(I) derivatives and gold nanoparticles in CRC therapy.

## 1. Introduction

Colorectal cancer (CRC) is the third most common type of cancer and has become one of the leading causes of death from cancers worldwide. CRC develops as a result of the change in normal colonic epithelium to adenomatous polyps due to mutations that target oncogenes, tumor suppressor genes, and genes related to DNA repair mechanisms [1,2]. Depending on the origin of the mutation, colorectal carcinomas can be classified as sporadic (70%), inherited (5%), and familiar (25%). Regarding 70% of the total cases of CRC, tumor onset is closely related to age, certain diseases, and lifestyle. In line with this, most of the cases of CRC are detected in Western countries and its incidence is growing alarmingly due to inadequate dietary patterns, tobacco smoking, and sedentarism, among other factors [3].

Depending on the grade of local invasion depth, involvement of lymph nodes, and metastasization, CRC can be divided into five stages: 0, I, II, III, and IV, with stage 0 being the earliest and IV the most advanced one. Poor prognosis is directly related with an advanced stage, as are therapeutic approaches. Whereas a tumor at stage 0 is easily removed by surgical extraction, patients with stage II disease and beyond need a combination of surgery, chemotherapy, and/or radiotherapy to enhance the success of treatment [1,4].

As aforementioned, the choice of treatment in CRC depends on the tumor features, and often, a combination of surgical resection and chemotherapy is selected. First-line chemotherapy in advanced CRC with metastasis is based on the use of oxaliplatin in combination with other drugs, namely 5-fluorouracil, leucovorin, and capecitabine [1,5,6]. However, the efficacy of oxaliplatin is limited due to its systemic toxicity and tumor-resistance to treatment. Consequently, trying to reduce platinum drug resistance and discovering new antitumor agents are considered areas of future development.

*Cis*-diamminedichloroplatinum(II), commonly called cisplatin, was the first platinum derivative successfully used as an anticancer agent against a wide range of tumor types. The mechanism of action of cisplatin consists of interacting with DNA bases, which, as a consequence, triggers DNA damage, leading to cancer cell death. Despite its effectivity, the unspecific targeting of DNA results in severe side effects that include neuro- and nephrotoxicity, among others. In order to maintain the strong anticancer effect of this drug while reducing its toxicity, a wide panel of cisplatin analogues has been synthetized [7].

Oxaliplatin ((*trans*-R,R-cyclohexane-1,2-diamine)oxalatoplatinum(II)) is a third-generation platinum-containing drug active against some cisplatin- and carboplatin-resistant tumors [8]. It acts as an alkylating agent on DNA, forming platinated intra-strand cross-links between two adjacent guanine bases or two adjacent guanine–adenine bases [9]. The formed adducts are bulkier and more hydrophobic than those formed with cisplatin and carboplatin, being more effective at inhibiting DNA synthesis. Although oxaliplatin displays fewer side effects than its precursor cisplatin, it causes adverse reactions that narrow its therapeutic index. Oxaliplatin is moderately myelotoxic and causes peripheral neuropathy, in addition to nausea, vomiting, and diarrhea. The systemic cytotoxicity displayed by oxaliplatin, as well as the rest of the cisplatin analogues, is not surprising considering that DNA is ubiquitously present in cancerous and healthy cells. Therefore, other pharmacological targets have been investigated, based on specific proteins over-expressed or unique to cancer cells [10,11].

Major advances in the understanding of CRC biology have led to the development of novel molecular targeted therapies for CRC. This approach consists of targeting specific cancer biomolecules including genes and proteins, as well as extracellular compounds that contribute to cancer growth and survival. Therefore, this type of treatment blocks the growth and spread of cancer cells while reducing damage to healthy cells. However, since the proteome might differ between tumor types, before the application of targeted therapy, it is necessary to perform a test to identify the genes, proteins, and other factors actually present in the tumor in order to enhance the success of treatment [12]. Herein, we describe some of the most relevant targets of CRC.

Thioredoxine reductase. The thioredoxin system plays a key role in the regulation of redox homeostasis and is directly involved in cell survival. Thioredoxin (Trx) and thioredoxin reductase (TrxR) are the main components of the thioredoxin system, which is overexpressed in many cancer types, among them, CRC [13]. Therefore, both Trx and TrxR have been regarded as interesting targets for chemotherapy and numerous natural and synthetic inhibitors have been reported [14,15,16].

The proteasome system. The ubiquitin/proteasome system (UPS) is a molecular complex that constitutes the main proteolytic pathway of eukaryotic cells. UPS is involved in the regulation of basic biological processes such as cell growth, proliferation, the cell cycle, and apoptosis [17] and deregulation of these processes causes malignant transformations. Since an increase in proteasome activity has been observed in many cancer cells, including CRC [18,19,20], proteasome inhibitors are able to selectively trigger the cell death of tumor cells [21,22].

Cyclooxygenase 2. Cyclooxygenase (COX) is involved in the biosynthesis of prostaglandins. A growing body of evidence suggests that isoform COX-2 promotes colonic carcinogenesis and cancer cell proliferation by inhibiting cell apoptosis, enhancing angiogenesis and cancer stem cell formation, and facilitating an immune response shift in the tumor microenvironment [23]. Therefore, COX-2 can be considered a target molecule in the treatment of CRC [24,25].

Carbonic anhydrases. Carbonic anhydrases (CAs) catalyze the reversible hydration of carbon dioxide to produce bicarbonate and a proton. Expression of the isoforms CA IX and CA XII has been shown to promote tumor cell invasion and metastasis in CRC [26], whereas isoform CA IV inhibits CRC progression [27]. To date, targeting of CA IX has been shown to be a promising strategy for CRC therapy [28]. Furthermore, some COX-2 inhibitors might act as well as CA inhibitors, thus enhancing their clinical potential [29].

Nitric oxide. Nitric oxide (NO) is a ubiquitous, water-soluble, free radical gas, which plays key role in various physiological and pathological processes, including cancer. The role of NO in carcinogenesis and tumor progression depends on its concentration [30]. At a low concentration, NO promotes tumor progression and the disruption of NO regular production is related to cancer cell death [31]. Therefore, pharmacological inhibition of inducible nitric oxide synthase (iNOS) has been proposed as a novel chemotherapeutic approach for the treatment of CRC [32]. On the other hand, NO is also emerging as a potential anti-oncogenic agent [33] since, at high concentrations, it displays cytotoxicity on cancer cells and triggers apoptosis [34]. In line with this, the upregulation of iNOS might display a comparable clinical effect to its inhibition [35].

Epidermal growth factor receptor and vascular endothelial growth factor. The blockage of the growth of new blood vessels in tumors by the inhibition of the epidermal growth factor receptor (EGFR) and vascular endothelial growth factor (VEGF) has been successfully tested against CRC [36,37]. In this regard, studies on the molecular mechanisms involved in CRC development have proposed novel treatment targets for CRC. The monoclonal anti-VEGF antibody bevacizumab, as well as the monoclonal anti-EGFR antibody cetuximab, have been shown to be very effective in clinical trials both as single agents and in combination with other conventional drugs [1,5,38].

Finally, immunotherapy has also been proposed as a promising therapeutic strategy for CRC treatment. In order to overcome the ability of a tumor mass to control the immune response and avoid its elimination, checkpoint inhibitors have been developed. The mechanism of action of this drug family consists of targeting CTLA4, PD1, and PD-L1 with monoclonal antibodies in order to re-activate lymphocytes T and to restore their capacity to kill cancer cells [39]. In this way, cancer vaccines might enhance the immune response to tumor-specific antigens. This approach consists of the administration of a highly immunogenic antigen, e.g., DNA, mRNA, and peptides, among others, that stimulates T cells upon interaction with antigen-presenting cells [40]. Another promising immunotherapeutic option is based on the blockage of inflammation mediators such as interleukins 6 and 11, which are strongly related to cancer onset and development. Monoclonal antibodies that target the IL-6 and IL-6 receptor are some examples [41]. The combination of immunotherapy with traditional anticancer strategies, such as conventional chemotherapy and radiotherapy, has been proposed as a novel approach to enhance the efficacy of antitumor therapy along with a reduction of side effects [42].

## 2. Gold(I) Complexes

In the search for alternatives to platinum-based drugs in the treatment of colorectal cancer, gold derivatives represent an excellent starting point, since gold complexes have a long history in medicine dating back thousands of years [43], although their exploration as anticancer agents has emerged more recently.

There are many examples of gold(I) and gold(III) derivatives described as potential anticancer agents; however, most of them display limitations concerning solution stability under physiological conditions. In particular, gold(III) derivatives are easily reduced to gold(I) or gold(0) that results in a loss of activity. For this reason, we have focused in this review on the description of gold(I) derivatives. The main advantage of gold derivatives in comparison with the traditional platinum-based chemotherapeutic drugs remains their general lack of affinity with DNA. Instead, the mechanism of gold complexes appears to be multifaceted. There is some evidence that some selected protein targets, such as thioredoxin reductase or deubiquitinases, rather than nucleic acids, mediate the anticancer effects of gold derivatives [44,45,46,47,48]. As previously discussed, those proteins are overexpressed on tumor cells and the inhibition of their activity is thus lethal to cancer cells, although with a significantly lower effect on non-cancerous cells. As a consequence, the systemic toxicity of gold complexes is expected to be reduced when compared to the current chemotherapy based on platinum-containing drugs.

### 2.1. Phosphane Gold(I) Derivatives

**Figure 1:**
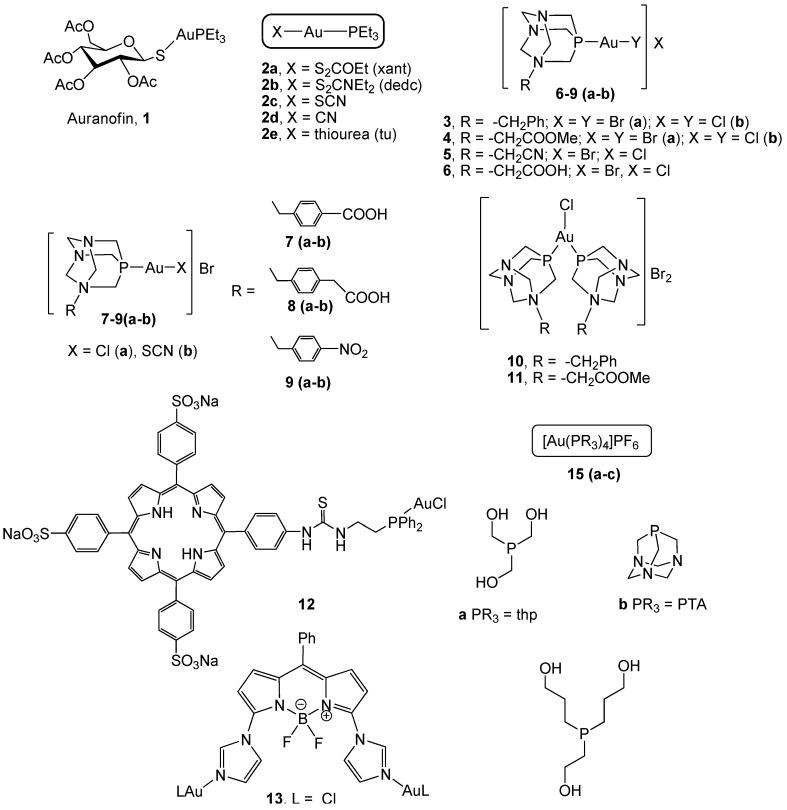
Gold(I) derivatives with monodentate phosphanes.

Auranofin (2,3,4,6-tetra-O-acetyl-b-1-D-thiopyranosato-S-(triethylphosphane)gold(I), 1, Figure 1) constitutes the first metal phosphane-based example introduced into clinical practice for chrysotherapy in the treatment of rheumatoid arthritis [49,50]. Apart from its use in rheumatoid arthritis, auranofin exhibits powerful antitumor activity in several in vitro and in vivo tumor models [51,52]. In addition, auranofin has entered, alone or in combination with other drugs, clinical trials due to its anticancer properties [53,54]. It is able to cross the cell membrane thanks to the lipophilic character of the triethylphosphane and although its mechanism of action is not clear, it is assumed to involve a fast displacement of the tetra-acetylthioglucose ligand that facilitates its further coordination to biological molecules. In vitro studies have revealed the ability of auranofin to overcome cisplatin resistance in human ovarian cancer cells, which confirms the assumption of a different mechanism of action from that previously described for cisplatin. In fact, auranofin has been identified as a potent inhibitor of both isoforms 1 and 2 (cytoplasmatic and mitochondrial, respectively) thioredoxin reductase (TrxR), thus causing an alteration of the redox state of the cell and inducing apoptosis [55,56]. This inhibition can be understood in terms of the high affinity exhibited by gold(I) to the redox-active selenocysteine residue of TrxR, and the consequent increase in reactive oxygen species (ROS) leading to an alteration of mitochondrial functions that initiates the apoptotic process. Interestingly, it has also been reported that treatment with low doses of auranofin (up to 1 μM) leads to a reduction in the expression level of TrxR2, which suggests that the effect of this drug on the redox balance is not limited to direct inhibition [57]. Although it was considered that auranofin displayed its anticancer effect by the inhibition of TrxR, further studies suggest that this compound might act as a multitarget mechanism of action. In line with this, treatment of cancer cells with auranofin has been found to be able to decrease the activity of the proteasome system by the inhibition of the activity of two 19S-associated deubiquitinases, namely UCHL5 and USP14 [46,58]. Therefore, other gold complexes might also interact with more than one cellular target, which would enhance their anticancer effect in comparison with the single target activity of platinum derivatives.

Similar derivatives to auranofin that maintain the AuPEt_3_ moiety [AuX(PEt_3_)], complexes 2a–e, Figure 1) have been described [59]. The thioglucose anion has been replaced with other mono-dentate ligands with different nucleophilicities: chloride, bromide, diethyldithiocarbamate, xanthate, thiocyanate, thiourea, and cyanate, in order to optimize their cytotoxic properties in addition to TrxR inhibition. Xanthogenate, cyanate, and thiocyanate gold(I) complexes appeared to be the most effective against colon carcinoma HCT-15 cell lines (with IC_50_ values of 0.61 µM, 2a; 0.32 µM, 2c; and 0.08 µM, 2d) and LoVo (IC_50_ values of 0.69 µM, 2a; 0.69 µM, 2c; and 0.67 µM, 2d), as well as against the multidrug-resistant subline LoVo MDR (IC_50_ values of 0.57 µM, 2a; 0.61 µM, 2c; and 0.65 µM, 2d). The presence of the [Au(PEt_3_)]^+^ moiety seems to be critical to achieving the antitumor activity. Moreover, TrxR seems to be the main target of these compounds, since all of them were able to inhibit both cytosolic and mitochondrial isoforms of isolated TrxR from rat livers; indeed, [Au(xant)(PEt_3_)] and [Au(dedc)(PEt_3_)] exhibited higher inhibition than that determined by auranofin. Their ability to inhibit TrxR depends on the additional ligand, based on the different binding strengths to the gold atom. Therefore, the halogens Cl and Br, which show scarce preference for gold(I), were found to be the least active in inhibiting thioredoxin reductase, whereas the rest of the ligands that are considered soft bases and exhibit a high preference for gold(I) are particularly effective at inhibiting thioredoxin reductases, especially [Au(dedc)(PEt_3_)] and [Au(xant)(PEt_3_)], probably by exchanging their ligands with the cellular nucleophiles when entering the cell.

Chloro gold(I) phosphane derivatives of the type [AuCl(PR_3_)] (PR_3_ = PMe_3_, PEt_3_, P^t^Bu_3_, PPh_3_) have exhibited significant antiproliferative effects against colon carcinoma HT-29, with IC_50_ values ranging from 4.2 to 5.2 µM, which are in the same range as auranofin (2.6 µM) and cisplatin (7 µM). The corresponding triphenylphosphane derivative displayed the highest cytotoxicity, which is in accordance with its highest lipophilicity that enhances its uptake into the cells [60].

The use of water-soluble *N*-substituted phosphanes derived from 1,3,5-triaza-7-phosphaadamantane (PTA) provided phosphane gold(I) complexes with a balanced relationship between liphophilicity and hidrophilicity (complexes 3–11, Figure 1) [61,62] that exert strong antiproliferative effects on human colorectal adenocarcinoma Caco-2 cell line clones PD7 and TC7 (from early and late passages, respectively). Complexes 3–6 with benzyl and the aryl substituents –CH_2_COOMe, –CH_2_CN, and –CH_2_COOH, displayed a comparable cytotoxicity (IC_50_ values of 4.26 µM, 4b; 4.51 µM, 5; and 6.79 µM, 6 in PD7 cells and 2.11 µM, 4a; 3.07 µM, 5; and 0.93 µM, 6 in TC7 cells) to that observed in auranofin (1.8 and 2.1 µM in PD7 and TC7 cells, respectively), and lower values than those found for cisplatin (37.24 and 45.6 µM in PD7 and TC7 cells, respectively), being able to induce apoptotic cell death [61]. Remarkably, the most water-soluble phosphane [PTA-CH_2_COOMe]Br gave the lowest IC_50_ values in both clones and, depending on the halogen, was lower for TC7 clones in the bromo compound 4a. A lower cytotoxicity has been exerted by the related derivatives [AuCl(PTA-R)]Br (complexes 7–9a, Figure 1) with *para*-substituted benzyl units in the PTA molecule (R = –CH_2_-*p*-COOH-C_6_H_4_, –CH_2_-*p*-CH_2_COOH-C_6_H_4_, and –CH_2_-*p*-NO_2_-C_6_H_4_) [62]. The replacement of the chlorine by thiocyanate (7–9b) gave higher IC_50_ values, probably due to the lower stability of the corresponding thiocyanate compounds in the solution.

The use of porphyrins in theragnosis – a field of medicine which combines specific targeted therapies with diagnostic tests – is widely known thanks to their rich photophysical properties that include: chemical and photochemical stability, high absorption coefficients and quantum yields, and emission in the therapeutic window (near-IR spectral region) [63]. Therefore, 5,10,15-tris(*p*-sulfonatophenyl)-20-(*p*-aminophenyl) porphyrin was transformed into an isothiocyanate that was coupled with [(2-(diphenylphosphino) ethylamine)-AuCl] to yield complex 12 (Figure 1) as a potential photosensitizer for photodynamic therapy (PDT) [64]. Given the potential implication of p53 for the success of chemotherapy, two colorectal cancer cell lines were selected: HCT-116 cells that express the wild-type p53 protein and SW480 that expresses an inactivated p53 protein. The compound displayed moderate cytotoxicity against both cell lines, with lower IC_50_ values on HCT-116 cells (56 µM) than on SW480 (72 µM), which might be indicative of the key role of p53 in gold complex-induced toxicity. Such cytotoxicity increased dramatically (up to four times) when the cells were illuminated for 30 min with white light, thus confirming the potential application of gold complexes as photosensitizers for the treatment of CRC.

The molecule BODIPY (4,4-difluoro-4-bora-3a,4a-diaza-s-indacene) is well-known as a promising fluorophore for imaging applications. Its combination with metallic derivatives is oriented to the development of metal-based drug candidates for theragnosis. Functionalization of BODIPY with two imidazole units has led to a bis-imidazole BODIPY moiety able to coordinate gold(I) units affording the homodinuclear complexes 13 and 14 (Figure 1) that are able to inhibit the proliferation of SW-480 colon cancer cells [65]. Their fluorescence properties allow the observation of intracellular localization of the complexes, which accumulated preferentially on mitochondria rather than on nucleus. This suggests that gold complexes might display less side effects than cisplatin due to the lack of interaction with DNA. Complex 13 also exerted very interesting anti-inflammatory properties, along with a low cytotoxicity, on Peripheral Blood Mononuclear Cells (PBMC). Given the key role of inflammation in tumor development, complex 13 might display a dual role in CRC therapy by the direct induction of tumor cell death and by the reduction of the pro-inflammatory status. However, further research on animal models is needed to validate this hypothesis.

Hydrophilic tetracoordinated gold(I) derivatives [Au(PR_3_)_4_]PF_6_ (15a–c, Figure 1) with the water-soluble phosphanes tris(hydroxymethyl)phosphine (thp), PTA (1,3,5-triaza-7-phosphaadamantane), and tris(hydroxypropyl)phosphine (thpp) revealed a structure-activity relationship (SAR) in HCT-15 human colon adenocarcinoma cells, with the most active being the derivative with the smallest phosphane (thp) (IC_50_ value of 13.21 µM for 15a *versus* 67.89 µM for 15c), via inhibition of the enzyme thioredoxin reductase activity [66].

**Figure 2:**
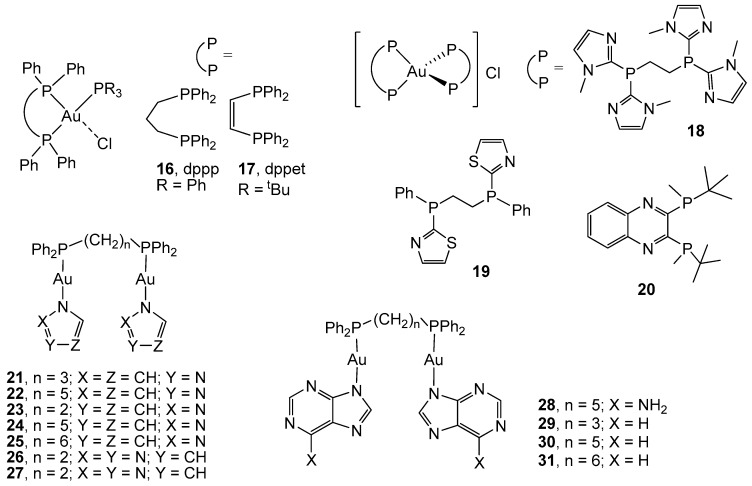
Gold(I) derivatives with bidentate phosphanes.

Four-coordinate Au(I) complexes containing disphosphane donor ligands have been reported as interesting antitumor agents, whose mechanism of action differs from that of cisplatin [67]. As an example, the complex [Au(dppp)(PPh_3_)Cl] (16, Figure 2) with the diphosphane 1,3-bis(diphenylphosphino)propane (dppp) has displayed anticancer activity in the micromolar range against an extensive panel of different types of cancer, among which four lines of colon cancer are included (IC_50_ values of 7.24 µM in COLO-205 cells, 4.68 µM in HCC-2998 cells, 4.17 µM on HCT-116 cells, and 5.50 µM in HCT-15 cells) [68]. Since the complex displayed significant toxicity in 29 of the 60 evaluated cancer cell lines, including those from colon cancer, the authors suggested that their effectiveness might depend on the tumor type. The presence of the diphosphane dppp and the chloride ligands confers the molecule intermediate lipophilicity character, thus avoiding increased side effects on mitochondria. The substitution of the triphenyl phosphane by the more basic and hindered tris(tert-butyl)phosphane (P^t^Bu_3_) and the introduction of the more rigid *cis*-1,2-bis(diphenylphosphano)ethene at the place of bis(diphenylphosphane)propane in complex 17 should induce changes in the physico-chemical properties of the final product [69]. Therefore, a higher cytotoxicity (IC_50_ = 0.29 µM) has been exerted by 17 in comparison with the related complex 16 in human colon cancer cell line HCT-116, thanks to a potent inhibition of TrxR and a subsequent increase in ROS levels.

Cationic bis-phosphane gold(I) compounds of the type [Au(PP)_2_]^+^ (PP = 1,2-bis-(diphenylphosphino)ethane and analogues) belong to the group of delocalized lipophilic cations (DLC) that are able to cross the cellular membrane and accumulate in mitochondria, leading to severe adverse effects in the case of highly lipophilic molecules [70]. In order to optimize the lipophilic-hydrophilic balance of the final derivatives, new azoyl-substituted diphosphane ligands, such as bis(dithiazol-2-ylphosphino)ethane and the asymmetric bis(1-methylimidazol-2-ylphenylphosphino)ethane, have been coordinated in gold(I) centres [71]. Compounds 18 and 19 (Figure 2), which display immediate lipophilicity (log*D*_7.4_ = 0.25 and 0.21, respectively) and consequently, an optimal balance of the lipophilic and hydrophilic nature, have been found to be active against human colon carcinoma HCT-116 (IC_50_ = 21.4 and 11.0 µM, respectively), with the most active being the complex with the symmetric bisphosphane. Studies of their mechanism indicated their capacity to inhibit the cysteine- (Cys) and selenocysteine- (Sec) dependent enzymes glutathione reductase (GR) and thioredoxin reductase (TrxR), respectively. The bis-chelated gold(I) bisphosphane bearing the phosphane 2,3-bis(tert-butyl(methyl)phosphino) quinoxaline (complex 20, Figure 2), has been described as a promising drug candidate in cancer therapy. This compound was a potent anticancer agent against different colon cancer cell lines (HT-29, SW-620, HCT-116, KM-12 and HCC-2998, with IC_50_ ≈ 0.5 µM), apart from a broad panel of other cancer lines [72,73]. Complex 20 selectively inhibited TrxR both in vitro and in cancer cells, without reacting with other Cys- or Sec-dependent targets, namely GR and GPx (Glutathione peroxidase), and significantly reduced tumor growth in several tumor xenografts in mice models of lung cancer without obvious hematologic toxicity after its treatment [73].

Raubaenheimer *et al*. [74] have described the activity of bisphosphane dinuclear gold(I) complexes with deprotonated N-donor molecules like imidazole, pyrazole, pyrrole, 1,2,3-triazole, 1,2,4-triazole, molecules 9H-purine, and adenine (complexes 21–31, Figure 2) against the CoLo colon cancer cell line. Compounds containing long aliphatic chains between the phosphorus atoms, dpppe (5C bridge between phosphorus atoms) and dpph (6C bridge), exhibited the highest cytotoxic potential, with IC_50_ values ranging from 0.005 to 0.039 µM, which are even higher than that observed for cisplatin (IC_50_ = 0.31 µM).

### 2.2. Gold(I) Complexes With S-Donor Ligands

It was also shown that phosphane gold(I) thiolates display a higher cytotoxicity than gold(I) thiolates and, in most of cases, than their chloride analogues, which indicates the importance of the combination of the ligands thiolate and phosphane in the same molecule for cytotoxic activity. The high stability conferred by thiolate ligands under physiological conditions could be a reason for the improvement of their effectiveness as cytotoxic derivatives. Furthermore, gold derivatives with tertiary phosphanes in the linear arrangement S-Au-P have been found to be highly active thanks to the lipophilicity of the phosphanes, which facilitates their transport across the membranes [75].

**Figure 3:**
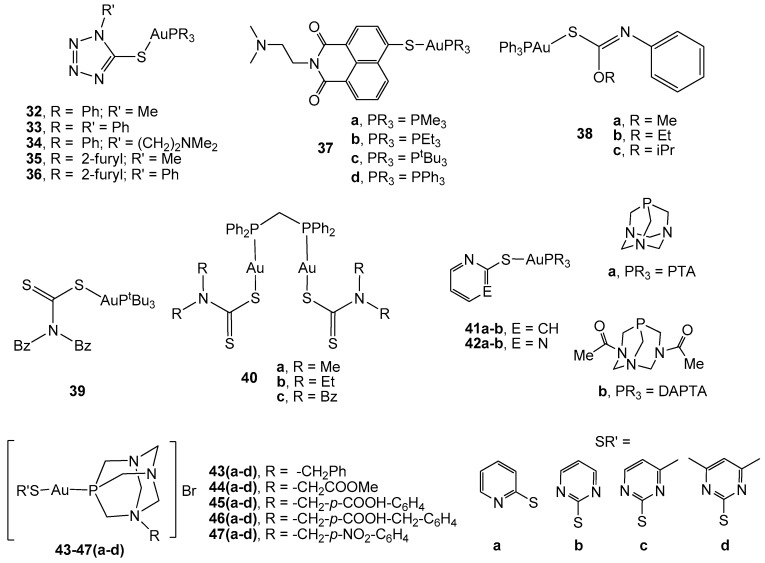
Thiolate phosphane gold(I) derivatives.

Therefore, gold(I) complexes with poly nitrogen-containing azoles (complexes 32–36, Figure 3) turned out be very effective in vitro inhibitors of the enzyme TrxR, in addition to displaying high cytotoxicity against HT-29 colon carcinoma cells (IC_50_ values ranging from 8.6 to 13.3 µM) [76]. The presence of triphenylphosphane, with a higher lipophilic character than the tri(2-furyl)phosphane, afforded an enhanced cellular uptake for the corresponding derivatives. This fact makes lipophilicity an important parameter for the cellular uptake of gold phosphane species.

The coordination of the gold(I) centre to naphthalimides, which are considered as bioactive ligands with significant cytotoxic activity [77], could lead to new agents with the ability to address multiple tumor targets. Therefore, a series of tionaphthalimide gold(I) phosphane complexes (37a–d) were described as active agents against HT-29 colon carcinoma, with IC_50_ values from 2.0 to 3.2 µM in the range of auranofin and lower than that observed for cisplatin [78]. However, no influence of the nature of the alkyl/aryl substituents on the phosphane was observed, since they did not have a strong impact on bioactivity. Regarding their mechanism of action, fluorescence microscopy imaging of the breast adenocarcinoma MCF-7 cell line showed a strong accumulation of the methyl derivative in the nuclei, as well as in other cell organelles. Moreover, the complex displayed strong antiangiogenic effects on a developing zebrafish embryo, which is a model that mimics tumor angiogenesis. Therefore, this complex might display a dual therapeutic approach for CRC treatment by avoiding the vascularization of the tumor mass together with its direct effect on the induction of cancer cell death.

Tiekink *et al.* [79,80] have demonstrated the influence of the substituents in triphenylphosphane gold(I) carbonimidothiates of the type [AuPPh_3_(SC(OR) = NPh)] (R = Me, Et, i-Pr) (compounds 38a–c, Figure 3). The three derivatives are cytotoxic against both 2D (HT-29 monolayer cells) and 3D (HT-29 cells spheroids) models of CRC, with the methyl partner being the most active (IC_50_ = 11.3 µM). Different apoptotic mechanisms could be delineated in the study. Complex 38a activated the p73 gene, while 38b and 38c activated p53. Complexes 38a and 38c showed significant enzymatic activity and a significant gene expression level on caspase-10 and induction of the up-regulation of BID expression in HT-29 cells. However, 38b induced an up-regulating effect on TNF (tumor necrosis factor) and TNFR (tumor necrosis factor receptor) genes in the cells, while 38a and 38c caused down-regulation of these genes. In addition, 38b also caused apoptosis by the JNK/MAP kinase pathway.

A series of mono and dinuclear phosphane gold(I) dithiocarbamate derivatives (compounds 39, 40a–c, Figure 3) have been described as active thiolate phosphane compounds against HCT-15 human colon cancer cells. There is a clear relationship between activity and structure, since the simplest dithiocarbamate skeleton (40a and 40b) afforded the most active compounds (IC_50_ = 9.53 and 11.97 µM, respectively, *vs* 29.67 µM for cisplatin) [81].

Although lipophilicity is important in the design of a drug, a balanced relationship between hydrophilicity and lipophilicity is also required so that it is water-soluble for its transportation and, at the same time, it should be able to pass through the phospholipid cell membrane. Accordingly, the use of water-soluble phosphanes, such as 1,3,5-triaza-7-phosphaadamantane (PTA) and 3,7-diacetyl-1,3,7-triaza-5-phosphabicyclo[3.3.1]nonane (DAPTA), afforded a series of highly water-soluble thiolate gold(I) complexes (solubility up to 120g/L) of the type [Au(SR’)(PR_3_)] (complexes 41–42a–b, Figure 3) that displayed a better cytotoxicity than that observed for cisplatin in WIRD colon cancer cell lines (LD_50_ ≈ 400 ng/mL *vs* 967 ng/mL for cisplatin) [82]. Similar thiolate phosphane gold(I) derivatives with *N*-substituted phosphanes derived from PTA (complexes 43–47a–d, Figure 3) [61,83] have been reported to be more cytotoxic than the corresponding halogen precursors against human colorectal Caco-2 cell lines. [Au(Spyrim)(PTA-CH_2_Ph)]Br (43b) was shown to enlarge the survival of athymic nude mice inoculated with HTC-116-luc2 cells and inhibit tumor growth without kidney and liver damage after gold treatment [84]. Complexes 45a–d, 46d, and 47a have demonstrated selective cytotoxic behaviour since they displayed viabilities of around 100% in a model of the intestinal barrier (differentiated Caco-2 cells), in addition to synergism with 5-FU when it is administered in combination with gold derivatives to cancerous cells. Therefore, the obtained results suggest that these complexes might be an interesting alternative to oxaliplatin in FOLFOX and related therapies. Preliminary studies on their mechanism have revealed the induction of apoptosis due to the increase in the intracellular ROS levels caused by the inhibition of TrxR activity [83].

**Figure 4:**
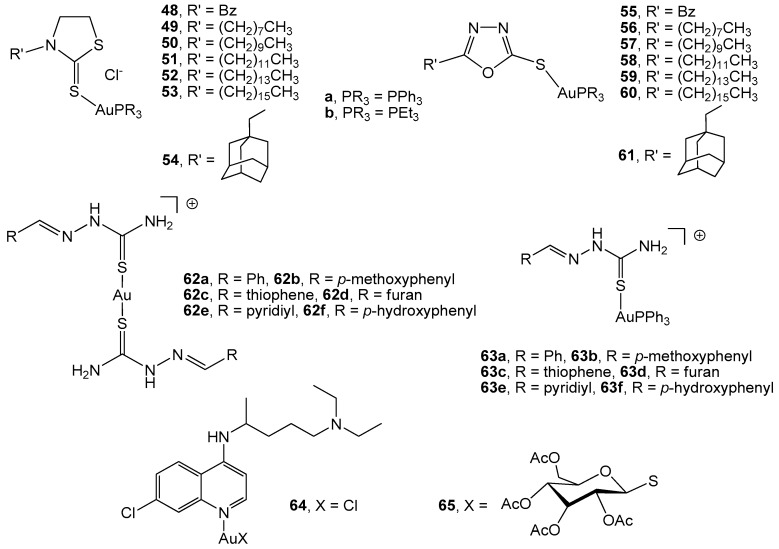
Gold(I) compounds with S-donor ligands.

The use of heterocyclic ligands with anti-inflammatory and analgesic activities biological properties, such as 3-Benzyl-1,3-thiazolidine-2-thione and 5-phenyl-1,3,4-oxazadiazole, has led to active gold(I) derivatives (complexes 48a,b and 55a–b, Figure 4) in murine colon cancer CT26-WT cells. Coordination to the AuPR_3_ moiety increased the biological activity in comparison with the ligands in the free form, with the most toxic compounds being the aryl phosphane (IC_50_ = 0.1 µM for 48a and 55a) compared to the alkyl phosphane (IC_50_ = 0.2 and 0.4 µM for complexes 48b and 55b, respectively) [85]. Thiazolidine and oxadiazole gold(I) derivatives with long hydrocarbon chains attached to the heterocyclic skeleton have been described and their biological activity on CT26-WT cells has been evaluated. Similar results to the corresponding benzyl counterparts (55a–b) have been obtained for the thiazolidine complexes (49–53a–b), while in the case of oxadiazole derivatives, the corresponding triethylphosphine 56–60b was more promising and selective than the triphenylphosphane counterpart 56–60a [86]. Using adamantane as the substituent R’ in the skeleton of both thiazolidine and oxadiazole molecules afforded high cytotoxic gold(I) complexes 54 and 61(a–b), which displayed high activity against TrxR. Complexes with thiazolidine were the most active in murine colon carcinoma CT26-WT (IC_50_ = 1.8 µM for 54a and 0.9 µM for 54b), probably due to a faster ligand exchange process in the presence of the selenocysteine of the thioredoxin reductase [87].

Aryl-thiosemicarbazones have also been described as molecules with biological properties such as nematocidal, insecticidal, antibacterial, antifungal, antiviral, and anti-inflammatory activities. Coordination to gold(I) (complexes 62a–f, Figure 4) [88] did not improve their anticancer activity in murine colon cancer CT26-WT cells; however, the presence of AuPPh_3_ units, as in complexes 63a–f, led to a slight improvement and inhibition of the TrxR activity. Drug-receptor docking analysis pointed to the occurrence of binding with Y116 and E30 residues, with Y116 being directly implicated in the enzyme catalysis. In consequence, the blockage of this amino acid might be a way to inhibit the enzyme activity.

A large number of chemotherapeutic drugs interact with DNA via covalent or non-covalent interactions. Platinum derivatives constitute the most extended examples of a covalent bond, leading to DNA adducts and activating various signal-transduction pathways. Gold(I) complexes do not usually interact strongly with DNA; however, the use of ligands able to produce DNA intercalation, for example, chloroquine, afforded the metallic complexes 64 and 65, which showed interaction with DNA. Both compounds induced growth inhibition in human colon carcinoma HT-29 and LoVo cell lines (IC_50_ = 30 µM) by two types of interaction with DNA, namely covalent binding through the metal centre, in addition to an electrostatic non-covalent interaction in the case of 64 and intercalation for 65, besides the inhibition of thioredoxin reductase [89].

### 2.3. Carbene Gold(I) Derivatives

N-heterocyclic carbenes (NHCs) have been considered as alternatives to phosphanes as ligands for Au(I) ions. Their strong σ-donor properties, together with the strength of metal NHC bonds and resistance to oxidation, lead to the formation of metal-NHC complexes with a high stability. These types of derivatives have emerged in the field of research of metallodrugs development. Consequently, many examples of carbene gold(I) derivatives have demonstrated their antitumor activity against different colon cancer cell lines. The relatively easy modification of the structure by changing the substituents has afforded a huge number of different molecules [90,91,92].

**Figure 5:**
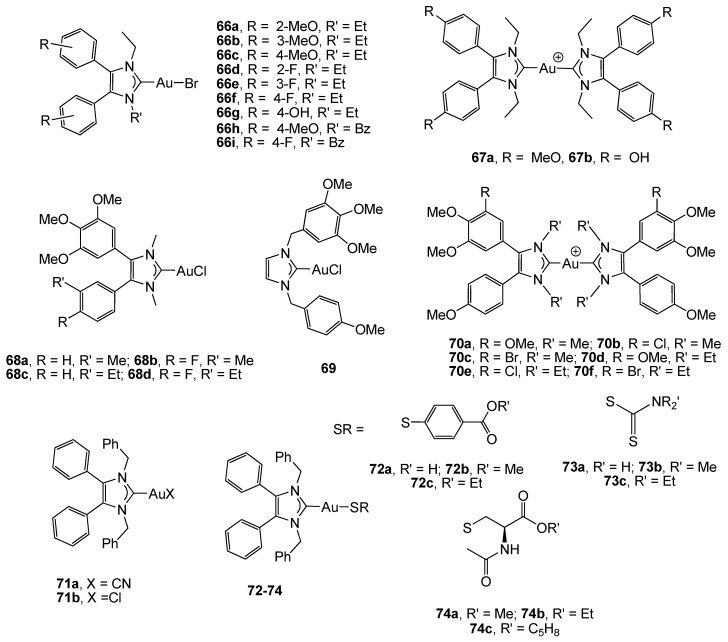
Carbene gold(I) derivatives based on the 4,5-diarylimidazol moiety.

Most of the described examples include structural modifications of a variety of 1,3-substituents at the imidazole core. However, some complexes with 4,5-diarylimidazoles (complexes 66a–i and 67a–b, Figure 5) have been described as potential antitumor agents against the HT-29 colon cancer cell line [93]. These new NHC gold halides caused growth inhibitory effects dependent on the substituents at the aromatic rings, since the 4-hydroxy group barely produced changes in the biological activity (IC_50_ = 17.0 µM), but methoxy and fluorine substituents significantly reduced the cytotoxicity (IC_50_ ranging from 2.3 to 4.3 µM similar to that found for cisplatin). All these compounds inhibited the TrxR, although the selective inhibition of the COX-1 enzyme by complex 66f opens a new perspective for the use of carbene gold complexes in medicinal chemistry. Cationic [bis(1,3-diethyl-4,5-diarylimidazol-2-ylidene)] gold(I) bromide complexes (67a–b) with similar carbene moieties to compounds 66a–i, have demonstrated considerable potential as new antitumor agents, with significant growth inhibition effects against colon (HT-29) carcinoma cell lines (IC_50_ ≈ 0.4 µM) [94].

Four chlorido-[1,3-dimethyl-4,5-diarylimidazol-2-ylidene]gold complexes 68a–d (Figure 5) were prepared and tested for cytotoxicity against a panel of different cancer cell lines. In order to evaluate structure–activity relationships, complex 69, which is the isomer of complex 68a, was also described. Both types of derivatives displayed a comparable cytotoxicity when applied to human colon carcinoma HT-29 cells (IC_50_ ranging from 11 to 34 µM), although they showed different pathways of accumulation in 518A2 melanoma cells [95].

Biscarbene gold(I) derivatives 70a–f with NHC ligands derived from the plant metabolite combretastatin (able to induce a rapid vascular shutdown in solid tumors) similar to the imidazole unit used in complexes 68a–d, have displayed a strong cytotoxicity against HT-29 (IC_50_ ranging from 0.06 to 0.16 µM) and HCT-116 (IC_50_ ranging from 0.07 to 0.30 µM) colon carcinoma cells, with the methoxy compounds 70a and 70d being the most active. These complexes showed an anti-angiogenic effect on different in vitro and in vivo models, although further assays on CRC animal models are needed to ensure their use in this kind of tumor [96].

N-heterocyclic carbene gold(I) complexes 71–74 (Figure 5) derived from the natural compound lepidiline A (1,3-dibenzyl-4,5-dimethylimidazolium chloride), which displays anticancer properties, were reported by M. Tacke et *al* [97]. Theoretical studies pointed to the advantages of employing an NHC ligand, as opposed to a phosphine, since calculated Au–Cl bond distances revealed a shorter bond in the NHC*-AuCl compound, and therefore, one that is stronger than in the related phosphane compound. The compounds were evaluated in the wild-type colon carcinoma HCT-116 cell line and in the p53 knockout mutant HCT-116^−/−^ in order to determine the role of p53 in the cytotoxic effect of the gold complexes. The results proved that the corresponding thiolate and dithiocarbamate derivatives (72-74) were more biologically active (IC_50_ ranging from 1.5 to 6.8 µM) than the compounds 71a-b without the Au–S bond (IC_50_ ranging from 1.5 to 6.8 µM). Moreover, a lack of p53 did not strongly influence the anticancer effect of the complexes, suggesting that this protein might not be related to their mechanism of action.

**Figure 6:**
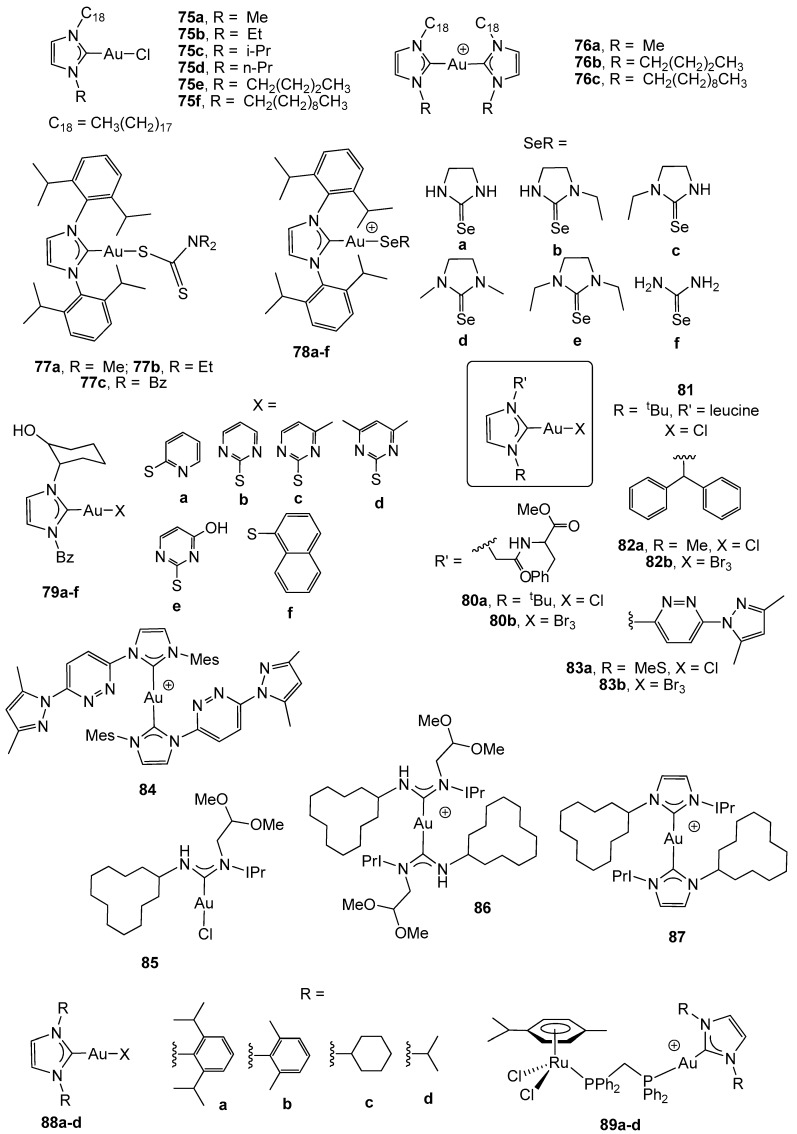
Structures of carbene gold(I) complexes.

Six N-heterocyclic carbene gold(I) derivatives with long aliphatic side chains have recently been reported by I. Ott *et al*. [98] and evaluated for in vitro cytotoxicity against HT-29 colon carcinoma. The metal-free imidazolium salts displayed good cytotoxic activity in the cancerous cells (IC_50_ ranging from 3.1 to 9.5 µM), which was not increased by the introduction of the metallic centre (complexes 75a–f, Figure 6) or even ameliorated in the biscarbene derivatives 76a–c (IC_50_ ranging from 9.08 to 38.6 µM). The cytotoxic activity was driven by the lipophilicity of the alkyl chains, giving rise to lower IC_50_ values in the case of the longest chain.

The use of bulky substituents in the imidazole ring, namely IPr = 1,3-Bis(2,6-diisopropylphenyl) imidazol-2-ylidene, has afforded carbene gold(I) derivatives with dithiocarbamate ligands (complexes 77a–c, Figure 6) that exhibited low anticancer activity on the HCT-15 human colon cancer cell line (IC_50_ ≈ 50 µM) [99]. Similar cytotoxicity (IC_50_ ranging from 33 to 51 µM *vs* 33 µM for cisplatin) has been found in complexes with selenones (78a–e) [100] or selenourea (78f) [101] instead of the dithiocarbamates against the same cancer cells. Docking studies revealed the presence of van der Waals interactions with the amino group in the TrxR enzyme.

A structurally diverse library of gold(I) and gold(III) NHC complexes (79–84, Figure 6) was described, and their inhibitory capacity against TrxR and antiproliferative properties towards the human colon adenocarcinoma HT-29 were evaluated, in order to stablish a structure-activity relationship (SAR) [102]. Most of the new compounds displayed a strong TrxR inhibition and led to antiproliferative effects in the range of established cytotoxic drugs, such as cisplatin and auranofin. Gold(I) NHC complexes were stronger TrxR inhibitors than gold(III) NHC complexes, the thiolate gold complexes 79a–f displayed the highest TrxR inhibition activity and a high cytotoxicity (IC_50_ ranging from 4.9 to 7.6 µM), and the insertion of a leucine-containing amino acid into the side chains at the NHC nitrogen atoms (complex 81) resulted in strong TrxR inhibition, although a low cytotoxicity (IC_50_ = 45.3 µM).

Two new N-acyclic gold(I) carbenes (NAC) (complexes 85 and 86) and the bis-N-heterocyclic carbene (NHC) (87) with the bulky substituents IPr and a relatively large hydrophobic ligand were tested as antiproliferative agents in human colon cancer HCT-116 cells. Complex 86 was highly active toward the colon cancer cell line (IC_50_ = 1.86 µM *vs* 25.52 µM for cisplatin), and only complex 85 was reacted with GSH (glutathione); this small molecule was selected in order to evaluate the interaction of complexes with protein-binding sites. When challenged against the model protein RNase-A, complexes 85 and 86 were able to form small quantities of a protein adduct, namely gold(I) ions directly bound to the protein with the loss of the carbene ligand [103].

Novel heterometallic RuAu carbene complexes (89a-d) have been found to be highly active in vitro against colon cancer HCT-116 cells, with a better cytotoxicity (IC_50_ values between 5.22 and 9.6 µM) than the monometallic gold counterparts (88a-d) (IC_50_ = 27.7–39.7 µM) [104]. Importantly, all these heterometallic RuAu complexes were also considerably less toxic to the non-tumorigenic human embryonic kidney cell line (HEK-293T) than the gold and ruthenium precursors.

**Figure 7:**
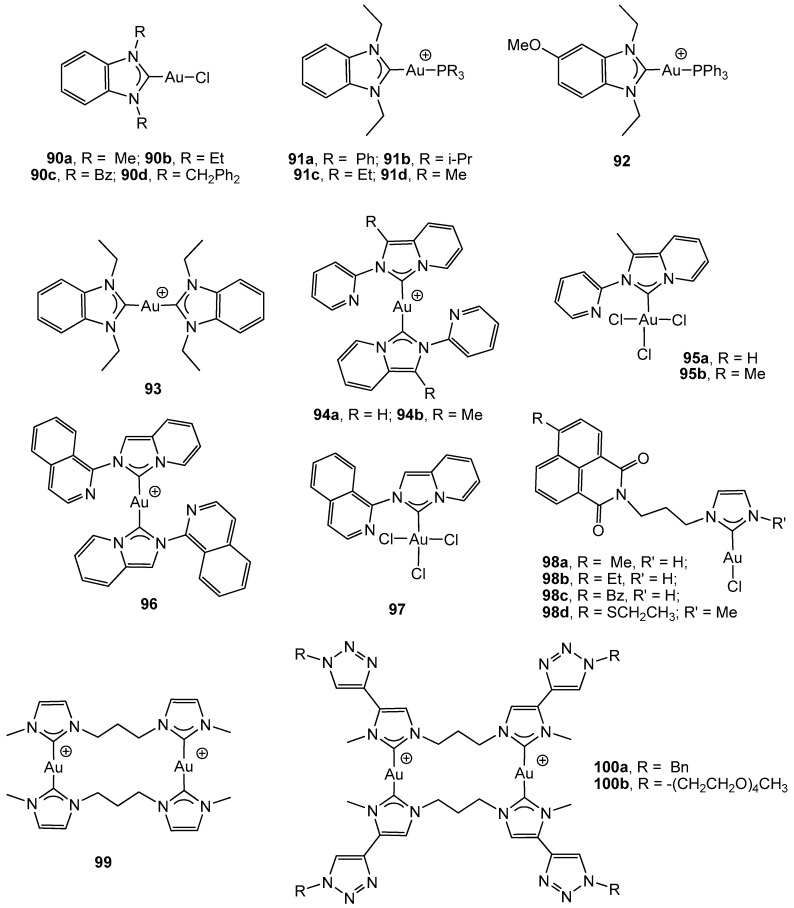
Structures of Au-NHC complexes.

A series of benzimidazol-2-ylidene gold(I) complexes was described and biologically investigated by I. Ott *et al.*, who focused on HT-29 and HCT-116 colon carcinoma cell lines [105]. Complexes 90a–d (Figure 7) displayed antiproliferative effects within a low micromolar range (IC_50_ ranging from 6.4 to 13.3 µM on HT-29 cells and in the range 6.7–24.6 µM on HCT-116 cells), which, in the case of 90b, were attributable to the induction of ROS formation [106], finally resulting in the apoptosis and necrosis of the cells due to damage of the mitochondrial integrity. The complexes were also able to more strongly inhibit the selenocysteine-containing enzyme TrxR than the structurally closely-related GR.

Substitution of the chlorine atom in complex 90b by different phosphanes has led to complexes 91a-d, which strongly inhibited the activity of the seleno-enzyme thioredoxin reductase (TrxR) and the zinc-finger enzyme poly(ADP-ribose) polymerase 1 (PARP-1) [107]. There was a clear dependence in TrxR inhibition on the size of the alkyl/aryl residues of phosphorus atoms. Therefore, decreasing the size of the residues of the phosphane ligand was accompanied by a significant increase in the efficacy of the enzyme inhibition. All the complexes induced antiproliferative effects towards human colon cancer cell line HT-29 (IC_50_ = 0.89–8.85 µM), which were correlated with the extent of cellular accumulation. Further studies [108] were conducted on complex 90b and also compared with the cationic complexes 91a and 93. The introduction of a positive charge in complexes 91a and 93 turned out to be a key feature that could increase the cellular uptake, induce mitochondrial accumulation, and improve general cytotoxic properties (IC_50_ ≈ 0.4 µM). The TrxR activity was efficiently inhibited by all three compounds in the order 90b > 91a > 93, revealing complex 91a (IC_50_ = 0.40 µM) as a promising compromise between good inhibitory effects against TrxR and strong antiproliferative/antimitochondrial properties.

A related cationic N-heterocyclic carbene derivative to complexes 91a–d (complex 92) induced apoptosis in colon cancer cells HT-29 (IC_50_ = 3.44 µM) by inhibiting thioredoxin reductase and inducing an crucial imbalance in cellular redox homeostasis [109]. It appeared to directly affect mitochondria by the rapid and irreversible inhibition of their respiratory activity. In addition, early signaling events associated with DNA damage have also been detected, thus pointing to DNA as an indirect target. All perturbations together lead to irreversible cell stress, with an accumulation of several persistent pro-apoptotic signals resulting in programmed cell death.

Gold(I) carbene compounds derived from pyridine annulated imidazole-2-ylidene, namely 1-methyl-2-(phenyl)imidazo[1,5-a]pyridine-2-ylidene and 2-pyridin-2-yl-2H-imidazo[1,5-a]pyridin-4-ylidene, were described, explored for their effect on human colorectal adenocarcinoma HCT-116, and compared with the corresponding gold(III) homologous complexes. In general, gold(I) complexes 94a–b displayed a higher cytotoxicity (IC_50_ values of 2.25 and 0.82 µM, respectively) than the Au(III) analogues 95a–b (IC_50_ values of 4.73 and 21.25 µM, respectively); however, higher IC_50_ values have been described for the methyl counterpart 95a in comparison with 95b [110,111]. All complexes inhibited cell growth through the induction of apoptosis. Additional molecular docking studies on complexes 94b and 95b with B-cell CLL/lymphoma 2 (BCL-2) and human topoisomerase I (Topol I) coupled with a DNA strand afforded complex 94b, which exhibited the highest affinity with BCL-2 and different binding affinities in both 94b and 95b complexes towards a DNA strand in a topoisomerase/DNA complex, which may interrupt the ability of the enzyme to hydrolyze the DNA phosphodiester bonds [110].

Similar annulated imidazole-2-ylidene compounds, with the naphthyl substituent 1-naphthyl-2-pyridin-2-yl-2H-imidazo[1,5-a]pyridin-4-ylidene, were also evaluated against the colorectal carcinoma HCT-116 cell line. Comparable results have been observed in complexes 96 (IC_50_ = 5.2 µM) and 97 (IC_50_ = 6.78 µM) to the homologous 94 and 95 complexes, with the corresponding gold(I) complex being more potent than the gold(III) 97 complex [112].

Incorporation of the napthalimide moiety in the N-heterocyclic carbene skeleton has led to the preparation of novel bioorganometallic anticancer agents with multiple nonrelated modes of action. Complexes 98a-d were found to interact with both DNA and the disulfide reductase enzyme thioredoxin reductase (TrxR) [113]. The complexes were potent DNA intercalators related to their naphthalimide partial structure and inhibited TrxR thanks to the incorporation of the gold(I) moiety. All complexes displayed efficient cytotoxic effects on HT-29 colon adenocarcinoma cells (IC_50_ = 1.9–4.9 µM), in addition to strong effects on tumor cell metabolism that affects respiration, cell impedance, and extracellular acidification.

Similarly, dinuclear di(N-heterocyclic carbene) gold(I) 99 and 100a-b and the related gold(III) complexes with bisiodine have been described and their antiproliferative effects towards HCT-116 cells among other cancer cell lines have been screened [114]. The di(N-heterocyclic carbene) ligands have a propylene linker between the carbene moieties and the imidazole backbone has been functionalized with a 1-benzyl- or 1-PEG-1,2,3-triazole ring (PEG = poly(ethylene glycol)). Higher antiproliferative activity has been observed for the functionalized complexes 100a–b (IC_50_ values of 2.8 and 0.26 µM, respectively), together with a higher selectivity towards cancerous cells with respect to healthy cells. Furthermore, complex 99 was unreactive with GSH (glutathione), whereas its related bisiodine-gold(III) reacted quickly to afford the corresponding gold(I) complex 99 and GSSG (glutathione disulphide).

### 2.4. Alkynyl Gold(I) Derivatives

**Figure 8:**
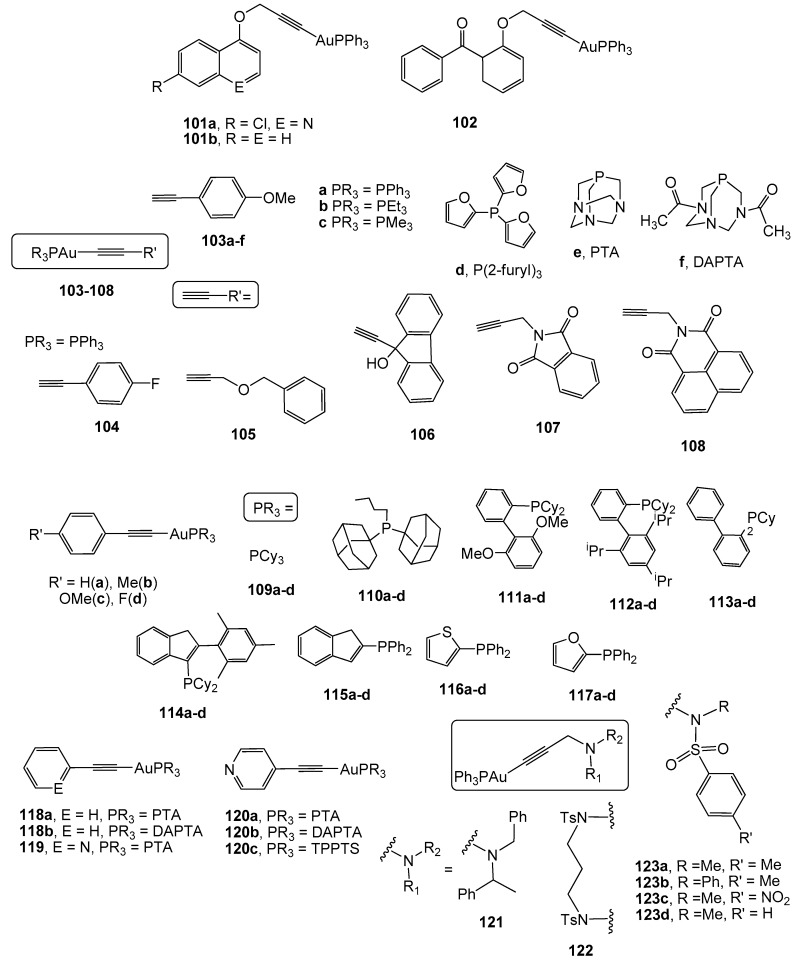
Alkynyl gold(I) derivatives.

The use of alkynyl ligands in the design of gold complexes with potential biological applications is quite recent [115] and, in the particular case of colon cancer, is less represented. Therefore, the first examples of alkynyl gold(I) derivatives tested against colon cancer were published in 2009 [116]. Complexes 101a–b and 102 (Figure 8) derived from the propargyl ethers 7-chloro-(4-propargyloxy)quinoline, 1-propargyloxynaphthalene, and 2-propargyloxybenzophenone, have been found to be active against the colon cancer cell line SW-480 (IC_50_ values of 9.3; 10 and 4.5 µM, respectively), although with higher IC_50_ values than that measured for cisplatin (IC_50_ = 3.5 µM).

Mononuclear alkynyl gold(I) derivatives with different alkyne molecules and different phosphanes as ligands (103–108, Figure 8) have been described in order to establish any SAR. Complexes with triphenylphosphane 103a–108 have shown strong antiproliferative activity against HT-29 colon carcinoma cells (IC_50_ values ranging from 1.6 to 12.0 µM). Remarkably, complexes with smaller alkynyl molecules displayed a faster uptake in these cells. These derivatives exhibited a high selectivity to the selenoenzyme thioredoxin reductase TrxR compared to the related enzyme GR, since a stronger inhibition of TrxR than that of GR was detected [117]. Further analyses involving tumor selectivity and mechanistic approaches related to protein kinases were conducted on complex 103a [118]. Its incubation with HT-29 colon cancer cells led to an activation of the mitogen activated kinases (MAPK) ERK1 and ERK2, which are involved in growth factor signaling and responsible for the regulation of biological functions such as cell growth, differentiation, and survival. The use of different phosphanes other than triphenylphosphane in complex 103 afforded compounds 103b-f that include the water-soluble molecules PTA and DAPTA [118]. All complexes exhibited high anticancer activity against HT-29 colon carcinoma, with 103b being the most active (IC_50_ = 2.6 µM). Moreover, they behaved as efficient inhibitors of thioredoxin reductase, with a linear correlation between the anticancer activity and the enzyme inhibition.

An extended study on related alkynyl derivatives with *para*-substituted phenyl moieties and different phosphanes (complexes 109–117(a–d), Figure 8), has revealed an implication of electronic and steric factors in the antiproliferative activity against HT-29 colon cancer cells (IC_50_ values ranging from 0.1 to 6 µM) and for the inhibition of thioredoxin reductase activity [119]. Therefore, the presence of strong electron-donating moieties in the ligand, which result in the delocalization of the metal active centre, has led to a high inhibition efficacy of TrxR activity, with complex 116c, with 4-methylphenylalkyne and thienyldiphenylphosphane as ligands, being the most efficient enzyme inhibitor in the inhibition of human colorectal carcinoma HT-29 cell proliferation.

The relevance of the alkyne structure has been highlighted in the study of the mechanism of action of complexes 118a and 119 (Figure 8). Complex 118a with the alkyne phenylacetylene displayed strong and selective antiproliferative activity against colon cancer cells Caco-2/TC7 (IC_50_ = 2.65 µM *vs* 37.24 µM for cisplatin), via inhibition of the enzyme TrxR, thus leading to an increase in ROS levels. The aberrant production of ROS induced an alteration of the balance between pro-apoptotic and anti-apoptotic proteins, loss of mitochondrial membrane potential, release of cytochrome c and, eventually, cell death by the mitochondrial apoptotic pathway [120]. However, the incorporation of a nitrogen atom in the phenyl ring (complex 119), namely 2-ethynylpyridine, led to different behaviour against this cell line [121]. Complex 119 barely inhibited TrxR activity; instead, it triggered TNF-induced necroptosis dependent on RIP-1 activation and NF-B signaling. Besides, complexes 120a–b, where the position of the nitrogen atom has been placed in a *para* position [122], exhibited small effects against tumor cell growth in HT-29 colon carcinoma (IC_50_ values of 56.09 and 74.78 µM, respectively) or even no cytotoxicity in the related 120c (with triphenylphosphane trisulphonated, TPPTS) [123].

A family of propargylic amine derivatives with triphenylphosphane (complexes 121–123, Figure 8) has been described by Bergamini *et al.* [124] as cytotoxic compounds against HT-29 human colorectal carcinoma. Their effectivity was dependent on the different substituents of the nitrogen atom. Therefore, the most effective at inhibiting cell growth were complexes 123a (IC_50_ = 7.9 µM) and 123d (IC_50_ = 11.0 µM) with the *para*-substituted benzene sulphonamide unit. In addition, complex 123a caused cell cycle arrest in the S phase on cancerous HT29 cells without the inhibition of TrxR activity.

**Figure 9:**
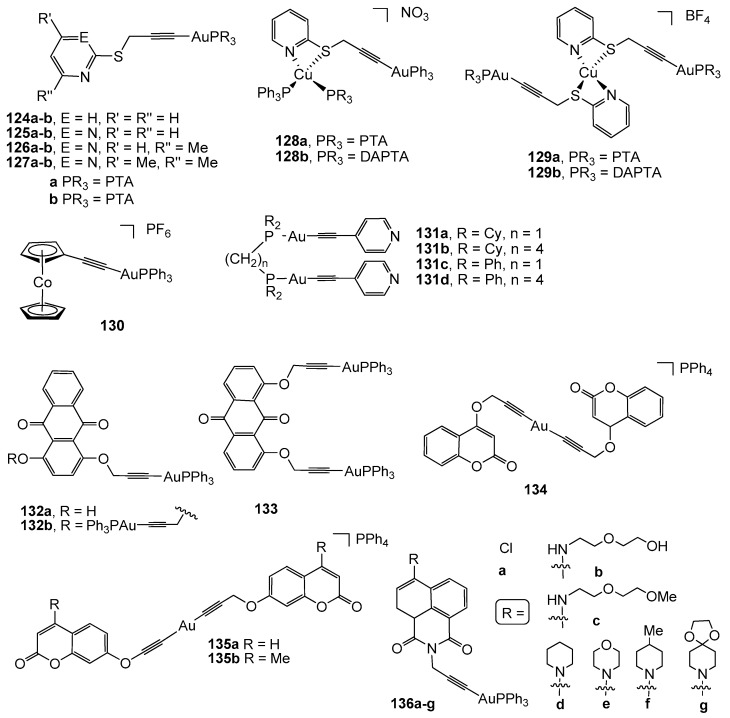
Mono and polinuclear alkynyl gold(I) derivatives.

A series of mono and polinuclear S-propargylthiopyridine derivatives have been reported as highly cytotoxic against human colon cancer cell lines Caco-2 clones PD7 and TC7. The corresponding mononuclear S-propargylthiopyridine and S-propargylthiopyrimidine (complexes 124–127a–b, Figure 9) [125,126] did not show an influence of the substituents of the alkyne moiety; nevertheless, a considerable difference in the antiproliferative activity was found, depending on the phosphane, with more active complexes being those with the PTA molecule (IC_50_ values ranging from 2.6 to 3.65 µM on PD7 clones and 2.7 to 4.5 µM on TD7 clones). Further administration of complex 124a to athymic nude mice xenografted with human HCT-116-luc colon cancer cells led to an increase in the mean survival time and life expectancy, in addition to moderate inhibition of the tumor growth without acute toxicity [126]. The incorporation of an additional metallic centre, namely Cu(I), afforded higher cytotoxic heterodinuclear and trinuclear complexes 128a–b and 129a–b, with the best IC_50_ value of 0.2 nM being obtained for the PTA compound 129a [125].

A cobalteceniumethynyl gold(I) complex (130) has recently been described as a cytotoxic compound against HT-29 colon carcinoma, thanks to its effectivity in inhibiting TrxR activity. Nevertheless, no selectivity was observed since its activity in a non-tumor cell line (RC-124 derived from a human kidney) was similar to that measured in the cancerous cells [127].

A family of dinuclear diphosphane gold(I) derivatives with 4-ethynylpyridine (complexes 131a–d, Figure 9) were screened for their antiproliferative effects on HT-29 colon carcinoma cells (IC_50_ values ranging from 0.6 to 15.3 µM) and their ability to inhibit TrxR activity. Only complexes with bis(diphenylphosphino)methane (dppm) and 1,4-bis(diphenylphosphino)butane (dppb) were described as active TrxR inhibitors, whereas the counterpart diphosphanes with cyclohexyl groups (complexes 131a–b) were found to be inactive against this redox enzyme [128].

The use of chromophore ligands has been proved to be an excellent strategy to study the visualization of drug cellular uptake and its biodistribution within the cell by fluorescent microscopy cell imaging. With this idea, several luminescent alkynyl gold(I) derivatives have been synthesized with mono- and dipropargylated dihydroxyanthraquinones, coumarins, and functionalized 1,8-naphthalimides. Therefore, complexes 132a–b and 133 were evaluated against LoVo colon adenocarcinoma, among other cancer cell lines, being less effective in the particular case of colon cancer. Their cellular uptake studies using cellular imaging revealed a significant accumulation of the complexes across the entire cytoplasm, including the organelles [129]. The corresponding luminescent complexes derived from coumarines functionalized by a propynyloxy group at the 4- or 7-position or two propynyloxy groups in the 6,7-positions (compounds 134 and 135a–b) have triggered strong cytotoxicity against HT-29 colon carcinoma (IC_50_ values of 1.84, 2.13, and 3.14 µM respectively), which was attributed to the cation tetraphenylphosphonium and their high inhibition of the enzyme thioredoxin reductase [122].

Complexes 136a–g (Figure 9) based on the functionalized 1,8-naphthalimide chromophores have been investigated for their cytotoxicity against several cancerous cells, including LoVo colon adenocarcinoma and human embryonic kidney HEK cell lines. Unfortunately, these complexes were more toxic to HEK cells; uptake studies on these cells and the protistan fish parasite *Spironucleus vortens* by confocal fluorescence microscopy showed their tendency for localization in mitochondria in HEK cells and in the hydrogenosomes in *Spironucleus vortens* [129].

## 3. Gold as a Vehicle: Biomedical Potential of Gold Nanoparticles in CRC Therapy

Conventional chemotherapy possesses several problems related to the poor oral bioavailability of the drugs, their non-specific distribution that leads to toxicity in healthy tissue, and the development of chemoresistance, among others. In an effort to increase the efficacy and safety of cancer therapy, during the past decades, researchers have focused on the design of novel ‘smart’ Drug Delivery Systems (DDSs). Unlike traditional DDSs, smart DDSs have the advantage of being able to differentiate between cancerous and non-cancerous cells in order to specifically target tumor cells, therefore leading to a controlled release of the chemotherapeutic drug at the tumor site [130,131,132]. In this regard, smart DDSs are able to target cancer cells by passive, active, and/or stimuli-responsive targeting. Passive targeting is a consequence of novel, leaky blood vessels derived from tumor angiogenesis along with poor lymphatic draining at the tumor site. As a result, nanoparticles in a size range from 10 to 100 nm are accumulated at the tumor site according to the so-called Enhanced Permeability and Retention effect (EPR) [132,133]. On the other hand, smart DDSs can be functionalized to target molecules that are overexpressed on cancer cells, which is known as active targeting [132]. Finally, stimuli-responsive targeting is based on the controlled release of drugs in response to intrinsic or extrinsic stimuli, including changes in the pH level, redox status, and temperature, among others [130].

Among all the different types of smart DDSs, gold nanoparticles (GNPs) have been of great interest in chemotherapy due to their unique physicochemical properties. GNPs are comprised of non-oxidized Au(0) atoms and can be synthetized in a wide range of structures, with nanospheres, nanorods, nanoshells, nanocages, nanostars, and nanocubes being the most used with biomedical purposes [130,134]. The customizable size and shape of GNPs is considered as one of their most interesting characteristics from a clinical point-of-view, since their morphology is closely related to their cytotoxic effect toward both cancerous and healthy cells. Cellular uptake of GNPs, which is mediated by endocytosis, is determined by their structure and therefore so is their biocompatibility, as well as their anticancer effect [135,136].

The small size of GNPs on a nanometric scale that allows them to pass through the cell membrane is also responsible for other features of great interest: a nanoparticle’s size is inversely proportional to its surface area, so GNPs display a large available surface area compared to volume ratio. This characteristic, together with the easy functionalization of their surface, allows the conjugation of GNPs with other molecules in order to improve several relevant parameters that will be herein discussed [134]. The aforementioned ability to functionalize GNPs is a consequence of the negative charge of their surface. Thiol and/or amine groups found in biomolecules and drugs are consequently able to interact with GNPs [134,137].

In this regard, the binding of chemotherapeutic drugs on GNPs has been proposed as a novel tool to overcome tumor resistance. When drugs are internalized by endocytosis, the efflux pump P-glycoprotein is no longer able to identify them and as a consequence, the drug is efficiently released into the cell and is able to reach its target [138]. However, this is not a unique way to revert multi-drug resistance. In the specific case of colorectal cancer therapy, Mercado-Lubo *et al*. [139] developed GNPs conjugated to the *Salmonella enterica* Typhimurium type III secreted effector protein SipA. This protein was previously reported to induce significant down-regulation in P-glycoprotein expression. As a result, SipA-GNPs displayed a dual effect on colon cancer cells. On one hand, when administered as single agents, they induced apoptotic cell death due to their intrinsic nature; on the other hand, they reversed resistance to doxorubicin, and the resulting drug cocktail successfully increased the anticancer effect of the individual ones.

Besides defeating chemoresistance, the conjugation of chemotherapeutic drugs to GNPs might also enhance their anticancer effect, as in the concrete case of phytic acid. The intake of this phytochemical has been positively correlated with a decreased incidence of CRC, although its application as a drug is limited due to its short lifespan. Arya *et al*. [140] reported that the functionalization of phytic acid on GNPs overcame the fast elimination of this bioactive compound and reduced the growth of CRC in a rat model. Moreover, the controlled release of chemotherapeutic drugs resulting in the functionalization of GNPs leads to a decrease in the side effects induced by platinum derivatives. Li *et al*. [141] designed a carbon nanobottle capped by GNPs in order to reduce the cytotoxicity of cisplatin. GNPs were covalently linked to the nanobottle by linkers sensitive to the unique conditions of the tumor microenvironment, and were thus able to be released by external stimuli, such as the low pH values that characterize tumor sites. Therefore, GNPs avoided the release of cisplatin until the nanomaterial reached the tumor site. As a result, these novel smart DDSs successfully killed colorectal carcinoma HCT-116 cells displaying a significantly lower toxicity in the human fetal lung fibroblast IMR-90 cell line.

Finally, modification of the GNPs’ surface might lead to increased tumor targeting. In previously discussed work, Arya *et al*. also included jacalin in their nanoformulation since this lectin recognizes the aberrant glycosylation patterns of the overexpressed colonic tumor antigens, thus leading to a more efficient approach of the nanomaterial to cancer cells. Similarly, Fan *et al*. [142] developed GNPs functionalized with the monoclonal antibody mAb198.3, which is able to bind FAT1. This surface protein is considered a marker of CRC due to its overexpression in colon cancer cells and therefore, the authors observed a marked reduction of the tumor mass in a mice model without evidence of significant systemic toxicity. In conclusion, the physicochemical properties of GNPs allow their functionalization in a wide range of molecules in order to increase their potential as smart DDSs.

### 3.1. Basis of Surface Plasmon Resonance in GNPs

Metals are characterized by the presence of free electrons in the conduction band. Therefore, when metal particles are exposed to light, a collective coherent oscillation of these free electrons in the particle surface is induced by the oscillating electromagnetic field of the light, forming dipole oscillation along the direction of the electric field of the light. The electromagnetic excitation is called surface plasmon polariton (a quasiparticle resulting from the coupling of the incidental electromagnetic waves and the dipole oscillation formed along the direction of the electric field of light) [143]. At certain frequencies, the amplitude of dipole oscillation reaches the maximum and a strong coupling of electromagnetic waves and the free electron oscillation occurs, and this phenomenon is called surface plasmon resonance (SPR) [144]. The SPR induces a strong absorption of the incident light and thus reflects non-absorbed frequencies, which can be detected using UV-VIS detection equipment. This phenomenon of resonance is at the maximum for noble metal (plasmonic) nanoparticles such as gold and silver, which makes GNPs promising theragnostic agents. Based on the SPR phenomenon, the potential of GNPs as contrast and photothermal agents for cancer diagnosis, monitoring, and treatment has been studied [143,145,146].

For spherical GNPs with a diameter smaller than 20 nm, the SPR phenomenon can be explained with the Mie theory applied to dipoles. The Mie model theoretically defines the loss of energy resulting from the scattering and absorption of the electromagnetic waves by the nanoparticles (Equation 1) [147]. Therefore, according to the Mie model, the plasmonic properties of GNPs are described after its extinction cross-section (C_ext_(λ)), which depends on the dielectric constant of the metal (ε), the dielectric constant of the non-absorbing surrounding medium (ε_m_), the wavelength of the incidental electromagnetic radiation (λ), and the particle size (R^3^). The ε(ω) is defined as the sum of the real (ε_r_) and the imaginary (ε_i_) part of the dielectric function of the material.

(1) $$C_{ext}\left(\lambda \right)=\frac{24\;\pi^{2\;}R^{3}\;\varepsilon_{m}^{3/2}}{\lambda }\;\frac{\varepsilon_{i}}{{\left(\varepsilon_{r}+2\varepsilon_{m}\right)}^{2}+\;\varepsilon_{i}^{2}}$$

The SPR occurs at the maximum C_ext_(λ), when the denominator is minimized and ε_r_ = −χ ε_m_, with χ being a geometrical factor (2 for spheres). Gold nanoparticles, in contrast with other metals with an SPR band in the UV region, show a strong SPR band around 520 nm [146]. However, the particle size and shape affect the SPR band, which is moved along the UV-visible-IR spectra depending on these features [145,148]. In spite of the fact that the total C_ext_(λ) is the addition of the absorption and scattering terms, for GNPs smaller than an incidental wavelength (d ≤ 20nm), the influence of absorption becomes more important and the photon energy transferred to the nanoparticle is rapidly converted to heat. On the other hand, for GNPs bigger than 70nm, scattering contribution prevails and photon propagation direction is modified [149,150].

In addition, the bandwidth, which is comprised of a radiative term and a nonradiative term, is also affected by size [151]. For small GNPs, the nonradiative term accounts for the biggest percentage, essentially producing heat, while for increasing size GNPs, the radiative term, and thus the scattering process, becomes more important [152].

Therefore, small GNPs are preferentially used as photothermal agents in cancer therapy, while GNPs with a higher contribution of the scattering term are preferable as contrast agents in photoimaging for diagnosis [153,154]. In conclusion, GNPs are promising theragnostic agents and by tuning their shape and size, their optical properties can be modified for use in both cancer treatment and diagnosis. 

### 3.2. GNPs as Contrast Agents

GNPs can be used as tumor biomarkers due to their optical properties derived from SPR and their capacity to specifically bind cancer cells, together with their small size and biocompatibility. This potential as contrast agents confers GNPs the ability to be used in diagnosis, as well as in treatment, easing tumor detection and delimitation when planning surgery and radiotherapy. Current contrast agents hold serious limitations, since iodine agents lead to short imaging times, occasional renal toxicity, and poor contrast. Studies run by Hanfield *et al.* [155] suggested GNPs as potential contrast agents which could overcome iodine restrictions. Quantitative pharmacokinetics in CD1 mice showed that GNPs are accumulated inside the tumor mass, obtaining a tumor:muscle gold ratio of 9.6 at 24 h after injection, enabling a clear delineation of the tumor for a longer time and with a higher resolution than iodine derivatives [155]. The improved retention time and contrast would permit the detection of tumors with a smaller size and providing better guidance in surgery or radiotherapy planification. Besides, toxicity tests showed that GNPs were cleared from the organism 30 days after injection and they did not have any adverse effect on mice, which survived for over a year with no sign of illness and toxicity in their organs [155].

GNPs are also being developed to be used in molecular imaging. With this purpose, GNPs are conjugated to different molecules, creating targeted GNPs which bind specific proteins in the cancerous tissue. Nguyen *et al.* [156] developed polyethylene glycol (PEG)-GNPs to enhance photoacoustic microscopy (PAM) and optical coherence tomography (OCT). Administration of PEG-GNPs to New Zealand white rabbits and Dutch Belted pigmented rabbits highly improved the image signal from the retinal and choroidal vessels and enabled the detection of individual blood vessels in the rabbits by both techniques, PAM and OCT [156]. Although, traditionally, computed tomography (CT) has not been considered as a molecular imaging modality owing to the disability of iodine agents to be conjugated with proteins, in the last years, targeted GNPs have been developed to enable imaging with this technique. Alric *et al.* [157] monitored the biodistribution of gadolinium chelate-coated GNPs in mice with magnetic resonance imaging (MRI) and X-Ray CT. The study confirmed that these nanoparticles could be used as contrast agents for MRI and CT, with MRI being more sensitive than X-ray imaging techniques [157]. Recently, GNPs have been developed to be used in dual mode techniques. In this regard, Liu *et al.* [158] described the synthesis and use of zwitterionic Au dendrimers bound to targeting arginine-glycine-aspartic acid peptide and 2,2’,2’’-(10-(2- (2,5-dioxopyrrolidin-1-yloxy)-2-oxoethyl)-1,4,7,10-tetraazacyclododecane-1,4,7-triyl) triacetic acid/Gd(III) complexes in enhancing dual mode CT/MRI. This application of GNPs was validated in a B16 lung cancer metastasis model, with similar results being obtained with both imaging techniques. Besides, contrast was intensified with the use of these GNPs, hence enhancing lung tumor CT and MR imaging [158].

In vitro and in vivo studies in colorectal cancer models have suggested the development of GNPs as promising contrast agents to be used in diagnosis. Tian *et al.* [159] synthesized Gd_2_O_3_ mesoporus silicananoparticles with GNPs with the purpose of amplifying MRI signals in SW480 human colon adenocarcinoma cells and male BALB/c mice. The results showed significant enhancement of contrast in MRI when silicananoparticles were administered together with GNPs and toxicity assays proved minimal cytotoxicity both in vivo and in vitro.

### 3.3. Gold Nanoparticles as Photothermal Agents

Photothermal therapy (PTT) is a non-invasive therapeutic approach to cancer treatment which consists of the generation of controlled hyperthermia from 41 °C to 50 °C on the tumor mass, without damaging the surrounding healthy tissues. For this type of therapy, the so-called photothermal agents, which are materials able to convert light into heat, are required [160,161]. In order to maximize the safety of PTT, photothermal agents must be located at the tumor site, but not on healthy tissue, since the thermal ablation threshold must only be reached on tumor cells. The selective accumulation of GNPs on tumor cells and their biocompatibility, along with other properties that will be discussed below, means that they are promising photothermal agents.

Whereas classic PTT restored the use of high-energy radiation to quickly heat photothermal agents and kill cancer cells, nowadays, near-infrared laser illumination is preferred. The main reason for this is that high-energy radiation triggered necrotic cell death, and the consequent inflammatory response led to undesirable side effects, whereas near-infrared light triggers apoptosis instead. Moreover, near-infrared light can safely and deeply trespass healthy tissue and reach the tumor-embedded photothermal agents. By modifying the size and shape of GNPs, their optical properties can be controlled to maximize the absorption of near-infrared light, thus allowing their application in the novel and safer PTT approach [160,162,163]. Furthermore, the tunable size and shape of GNPs is related to a higher photothermal conversion efficiency [162,164]. Finally, the easy surface functionalization of GNPs allows their use as dual photothermal and chemotherapeutical agents [165], which in consequence, leads to a more efficient reduction of the tumor mass. In this regard, it has been reported that GNP-mediated PTT sensitizes tumor cells to other drugs [162], thus reducing their necessary dose or even overcoming drug resistance. Emami *et al*. [166] functionalized GNPs with anti-Programmed Death-Ligand 1 (PD-L1) antibodies, which are overexpressed in CRC cells, in order to minimize the entrance of the nanoparticles in non-cancer cells. Furthermore, the authors conjugated doxorubicin to anti-PD-L1-coated GNPs and performed both near-infrared PTT and chemotherapy on CT26 murine colon cancer cells. As a result, the uptake of doxorubicin was significantly improved and strong rates of cell death were reported.

In line with this, the development of novel GNP-based nanocomplexes with a dual photothermal and chemotherapy role is an upward trend for the treatment of CRC. Wang *et al*. [24] designed an in situ hydrogel in which the following were incorporated: a) polyethylene glycol-coated gold nanorods and b) paclitaxel nanocrystals coated with a P-glycoprotein inhibitor, in order to reduce multidrug resistance. The aim of this research was the development of a long-acting therapeutic approach to combine the benefits of GNP-mediated PTT and chemotherapy. Assays performed in a mice model of CRC showed that the combination of PTT and chemotherapy resulted in a great reduction of the tumor mass when compared to the administration of paclitaxel as a single agent. No evidence of side effects was noticed, which suggests a good biocompatibility, and tumor recurrence was significantly lower than in mice which just received PTT or chemotherapy alone. Similarly, Chuang *et al*. took advantage of the unique properties of GNPs to develop a novel nanocomplex with a dual photothermic and chemotherapeutic role. The authors encapsulated gold nanorods and doxorubicin in poly(lactic-*co*-glycolic acid) (PLGA) nanoparticles. The aim of the encapsulation in PLGA was to enhance the uptake of doxorubicin. Therefore, once GNPs received near-infrared light, the resultant heat would destroy PLGA, thus leading to a controlled release of the drug at the tumor site. Assays performed in a mice model of CRC showed that the combination of photothermal and chemotherapy resulted in a significant reduction of the tumor mass and no toxicity in healthy tissue was noticed. In summary, the capacity of GNPs to turn light into heat makes them a key element in novel CRC therapies.

The main limitation of classic PTT remains its incapacity to destroy cancer cells located outside the irradiation area, which might lead to tumor recurrence. Similarly, the treatment of disseminated metastasis might be compromised. However, the use of GNPs as photothermal agents has been proposed as a novel approach to overcome this disadvantage, since GNPs penetrate also metastatic nodes [162,167]. Nam *et al*. [168] observed a significant elimination of residual tumor cells in a bilateral mice model of CT26 colon carcinoma, which were significantly lower upon treatment with a combination of GNP-mediated PTT and doxorubicin, thus leading to high survival and low recurrence rates. The authors reported that treatment with photothermal and chemotherapy resulted in an enhanced immune response that might be responsible for the reported effect on disseminated cells.

In addition to the aforementioned positive effect on the immune response, the role of GNP-based PTT in the treatment of metastasis is closely related to the previously discussed theragnostic potential of GNPs. GNP-mediated imaging might provide guidance for PTT and thus eradicate disseminated cells along with the located tumor mass, which might reduce recurrence rates. The combination of imaging and PTT has been successfully evaluated in a mice CRC model by Mulens-Arias *et al*. [169] by photoacoustic imaging and photo-ablation. Therefore, the production of spherical gold nanoparticles in one-pot by using polyethyleneimine (PEI) as a reducing agent at a molar ratio [PEI]:[Au^3+^] of 5 led to more compact aggregates, exhibiting a fractal-like lattice which favored plasmon coupling and NIR absorption.

### 3.4. Gold Nanoparticles in Photodynamic Therapy

Photodynamic therapy (PDT) is a treatment based on the use of light-activated drugs called photosensitizers. Upon the injection of photosensitizers in cancerous tissue, the tumor is irradiated with specific wavelengths, hence exciting the photosensitizing agents, which leads to energy transfer that generates ROS and, eventually, cell death. Based on their capacity to target cancerous cells and accumulate in the tumoral tissue, different in vitro studies have suggested the use of GNPs as photosensitizer carriers for PDT in colon cancer treatment.

Jeong *et al*. [170] synthetized thiol-protected GNP covalents conjugated to the photosensitizer Toluidine Blue O and in vitro assays in SW480 human colon adenocarcinoma cells suggested the use of GNPs as conjugates to deliver photosensitizers into the cancerous cells. Simon *et al.* [171] developed pluronic-stabilized 3-D close-packed nanoassemblies of gold nanoparticles loaded with methylene blue. The photosensitizing capacity of the GNPs was determined by measuring their efficiency in singlet oxygen generation (^1^O_2_), obtaining a much higher rate of ^1^O_2_ generation than free methylene blue. Besides, performing PDT sessions on GNP-treated C26 murine colon carcinoma cells showed augmented anticancer effects in comparison with free methylene blue-treated C26 cells [171]. Obaid *et al.* [172] compared the influence of different targeting molecules on PDT in GNPs stabilized by the photosensitizer zinc phthalocyanine disulphide (C11Pc) and PEG. The results showed that functionalized GNPs, either with jacalin, which is a lectin able to bind the cancer-associated Thomsen-Friedenreich (T) carbohydrate antigen, or monoclonal antibodies specific for human epidermal growth factor receptor-2, could mediate a selective photodynamic treatment response in HT-29 colon cancer cells [172].

However, although different clinical trials have showed promising results when using GNPs in PDT against certain cancers, such as bladder, skin, lung, esophagus, and cervix cancer [173,174,175,176], no in vivo studies or clinical trials have showed solid results to support the use of PDT in colorectal cancer treatment. One feasible reason seems to be the limited tissue penetration of light, which prevents the excitation of photosensitizers in the colorectal region. To overcome PDT limitations, radiation with a higher penetrance than wavelengths corresponding to UV-visible-NIR spectra, such as X-ray, are needed.

### 3.5. Gold Nanoparticles as Radiosensitizers

Despite the effectiveness of radiotherapy (RT) at reducing a tumor mass, this treatment induces toxicity in healthy tissues, leading to a worsening of the patient’s quality of life. Side effects of RT, which range from gastrointestinal discomfort to dermatitis [177,178,179], restrict the dosage and might even lead to an interruption of the treatment. Therefore, the use of radiosensitizers is emerging as a novel adjuvant treatment for RT due to the decrease in systemic cytotoxicity caused by the reduction of the necessary dose to kill cancer cells. In this context, the potential of GNPs to increase tumor cell radiosensitivity has been widely studied.

The mechanism by which GNPs increase the effectivity of RT is not fully understood yet, although two main hypotheses have been proposed. Firstly, materials with a high atomic-number (*Z*) such as gold (*Z* = 79) display increased photoelectric photon absorption in the kilovoltage energy range. Therefore, cells that have internalized GNPs would absorb more energy per unit mass than the ones that have not incorporated nanoparticles [180,181]. In other words, according to the previously discussed preferential accumulation of GNPs in tumor tissue, the uptake of GNPs is directly related to an increase in radiosensitivity due to the physical properties of gold atoms. On the other hand, some authors have reported that GNPs lead to the inhibition of the enzyme thioredoxin reductase. Since the toxicity of RT is mediated by an aberrant production of ROS, authors have suggested that the inhibition of TrxR mediated by GNPs leads to a failure in the cell detoxification systems and a subsequent improved response to RT [182,183]. These two hypotheses are not exclusive and the evidence obtained so far suggests that both can occur simultaneously, which might explain the radiosensitizer effect of GNPs.

In the specific case of CRC, evidence which supports the potential of GNPs as radiosensitizers has been obtained, although the concrete mechanism of action has not been fully explained. Hau *et al*. [184] found that GNPs increased the sensitivity of LoVo human colon cancer cells to both kilo and mega-voltage radiation. Similar results were further obtained by Saberi *et al*. [185] for human colorectal adenocarcinoma HT-29 cells irradiated with X-ray mega-voltage energy. Interestingly, the authors reported that the radiosensitizer effect was observed even at non-toxic concentrations. Furthermore, GNPs can be combined with other techniques in order to enhance the effectivity of RT, such as electroporation. In this regard, Rezaee *et al*. [186] observed that the concomitant use of electroporation and GNPs synergistically increased the sensitivity to mega-voltage photon energy in HT-29 cells. Moreover, the authors found that the observed synergistic effect of GNPs, electroporation, and ionizing radiation was tumor-dependent, since a lower response to combined treatment was found in the non-cancer CHO cell line (derived from a Chinese hamster ovary).

Although bared nanoparticles have been employed in the aforementioned research, the functionalization of GNPs, together with their synergistic effect with RT, has opened the door to the development of novel combinations of radiation and chemotherapy of great efficacy, along with safety. Mirrahimi *et al*. [187] developed a novel nanocomplex composed of cisplatin and GNPs co-loaded on an alginate hydrogel network which, in combination with X-ray mega-voltage energy, decreased CT26 colon adenocarcinoma cell viability to almost 95%.

The findings of Kim *et al*. [188] are also noteworthy due to the great importance of the re-acquisition of radiosensitivity on hypoxic tumors. As previously cited, the efficacy of RT lies in the generation of ROS that damage DNA and eventually trigger cancer cell death. However, those areas of solid tumor located farthest from blood vessels are able to survive in a low concentration of oxygen, namely hypoxia. Oxygen deficiency avoids the production of ROS triggered by RT, and as a consequence, limits its clinical application to solid tumors [189]. However, the promising results obtained by Kim *et al*. [188] suggest that GNPs might overcome this limitation, since they observed that non-toxic concentrations of GNPs increased the response to radiation in a mice model of CRC and successfully reduced the tumor mass. Cell culture assays in the mouse colon cancer CT26 cell line showed that the combination of GNPs and radiation under hypoxic conditions led to an increase in ROS production. In conclusion, the radiosensitizer effect of GNPs might increase the range of RT applications to include hypoxic tumors.

## 4. Conclusions

Despite the abundance of different therapies used to treat CRC, it remains one of the most aggressive cancers, with a poor prognosis and a high death rate. Platinum compounds, mainly oxaliplatin, have been traditionally used in chemotherapy against CRC. However, due to the significant side effects of platinum-containing drugs, along with tumor resistance to treatment, new metal-based compounds have been developed and the data collected and exposed in this work suggest that gold could be a promising alternative in the future. Firstly, gold(I) compounds avoid cross-resistance to platinum derivatives due to their lower affinity for the DNA strand. Instead, gold complexes interact with proteins that are overexpressed in tumor cells, such as TrxR or the proteasome system. Therefore, gold(I) compounds display a higher selectivity for a tumor mass than non-cancerous cells. In addition, the role of gold in CRC treatment is not limited to gold(I) complexes, since the use of GNPs has been shown to enhance the effectiveness of classic therapeutic approaches. In this regard, GNPs can be used in different therapies, such as PTT, RT, or PDT, and act as carriers to deliver the drugs into the tumoral tissue, as well as being used in diagnosis.

## Abbreviations

PP	1,2-bis-(diphenylphosphino)ethane
PTA	1,3,5-triaza-7-phosphaadamantane
IPr	1,3-Bis(2,6-diisopropylphenyl) imidazol-2-ylidene
dppb	1,4-bis(diphenylphosphino)butane
DAPTA	3,7-diacetyl-1,3,7-triaza-5-phosphabicyclo[3.3.1]nonane
BCL-2	B-cell CLL/lymphoma 2
dppm	Bis(diphenylphosphino)methane
CA	Carbonic anhydrase
CRC	Colorectal cancer
CT	Computed tomography
COX	Cyclooxygenase
Cys	Cysteine
DLC	Delocalised lipophilic cations
dppp	Diphosphane 1,3-bis(diphenylphosphino)propane
DDS	Drug delivery system
EPR	Enhanced permeability and retention
EGFR	Epidermal growth factor receptor
GSH	Gluthathione
GSSG	Glutathione disulphide
GPx	Glutathione peroxidase
GR	Glutathione reductase
GNP	Gold nanoparticle
iNOS	Inducible nitric oxide synthase
MRI	Magnetic resonance imaging
MAPK	Mitogen activated kinases
NAC	N-acyclic carbene
NHC	N-heterocyclic carbene
NIR	Near-infrared
NO	Nitric oxide
OCT	Optical coherence tomography
PAM	Photoacoustic microscopy
PDT	Photodynamic therapy
PTT	Photothermal therapy
PARP-1	Poly(ADP-ribose) polymerase 1
PEI	Polyethyleneniumine
PEG	Polyethylene glycol
PLGA	Poly(lactic-co-glicolic acid)
PD-L1	Programmed death-ligand 1
RT	Radiotherapy
ROS	Reactive oxygen species
Sec	Selenocysteine
SAR	Structure-activity relationship
SPR	Surface plasmon resonance
Trx	Thioredoxin
TrxR	Thioredoxin reductase
thp	Tris(hydroxymethyl)phosphine
thpp	Tris(hydroxypropyl)phosphine
TPPTS	Triphenylphosphane trisulphonated
TNF	Tumor necrosis factor
TNFR	Tumor necrosis factor receptor
(UPS)	Ubiquitin/proteasome system
VEGF	Vascular endothelial growth factor
C11Pc	Zinc phthalocyanine disulphide
